# 

**Published:** 1906-12

**Authors:** 


### The Library of the Bristol Medico-Chirurgical Society.

*The following donations have been received since the publication of the List in September:*

*November 30th, 1906.*

| | |
|---|---|
| John Beddoe, M.D. (1) | 5 volumes. |
| F. H. Edgeworth, M.B. (2) | 9 ,, |
| L. M. Griffiths (3) | 5 ,, |
| Middlesex Hospital (4) | 1 volume. |
| Scholastic Trading Depôt (5) | 2 volumes. |
| R. Shingleton Smith, M.D. (6) | 1 volume. |
| The Surgeon-General, United States Army (7) | 1 ,, |

### SIXTY-SECOND LIST OF BOOKS.

The titles of books mentioned in previous lists are not repeated.

The figures in brackets refer to the figures after the names of the donors, and show by whom the volumes were presented. The books to which no such figures are attached have either been bought from the Library Fund or received through the *Journal*.

**Allbutt and H. D. Rolleston, T. C.** [Eds.] *A System of Medicine.* Vol. II. Part I. ... [2nd Ed.] 1906

**Armstrong, J.** ... *Poetical Works* ... (3) [1796]

**Atkinson, S. B.** ... *Golden Rules of Medical Evidence* ... [N.D.]

**Bland-Sutton and A. E. Giles, J.** *Diseases of Women* ... 5th Ed. 1906

**Bramwell, B.** ... *Clinical Studies.* Vol. IV. ... (6) 1906

**Brown and W. T. Ritchie, J. J. G.** *Medical Diagnosis* ... 5th Ed. 1906

**Brunton, Sir L.** ... *Collected Papers on Circulation and Respiration.* (1st Series) 1906

**Carter, A. H.** ... *Elements of Practical Medicine* ... 9th Ed. 1906

*Catalogue (Index-) of the Library of the Surgeon-General's Office, U.S. Army* 2nd Series. Vol. XI. (7) 1906

**Cooper, R. H.** ... *X-rays in General Practice* ... 1906

**Cornaro, L.** ... *Sure Methods of attaining a Long and Healthful Life.* (Translated) (2) 1829

**Crook, H. E.** ... *High Frequency Currents* ... 1906

*Davos as Health Resort* ... 1906

**Dixon, W. E.** ... *A Manual of Pharmacology* ... 1906

**Dowse, T. S.** ... *Lectures on Massage and Electricity* ... 6th Ed. 1906

*Encyclopedia and Dictionary of Medicine and Surgery.* Vol. II. ... ... ... [1906]

**Forel, A.** ... ... *Hypnotism.* (Tr. from 5th German Ed. by H. W. Armit) ... ... ... ... ... ... ... ... ... ... 1906

**Freyer, P. J.** ... *Enlargement of the Prostate* ... ... ... ... 3rd Ed. 1906

**Gee, S.** ... ... ... *Auscultation and Percussion* ... ... ... ... 5th Ed. 1906

**Giles, J. Bland-Sutton and A. E.** *Diseases of Women* ... ... 5th Ed. 1906

**Handley, J.** ... ... *Mechanical Essays on the Animal Oeconomy* ... (3) 1721

**Handley, W. S.** ... *Cancer of the Breast* ... ... ... ... ... ... ... ... 1906

**Jones, H. L.** ... *Medical Electricity* ... ... ... ... ... ... ... 5th Ed. 1906

**Marshall, C. F.** ... *Syphilology and Venereal Disease* ... ... ... ... ... ... 1906

**Martindale and W. W. Westcott, W. H.** *The Extra Pharmacopœia* 12th Ed. 1906

**Miles, A. Thomson and A.** *Manual of Surgery.* Vol. I. ... ... 2nd Ed. 1906

**Moore, G.** ... ... *Nose and Throat Diseases* ... ... ... (3) 3rd Ed. [N.D.]

**Morrison, J.** ... *Medicine no Mystery* ... ... ... ... ... ... ... (2) 1829

**Moynihan, B. G. A.** *Retro-peritoneal Hernia* ... ... ... ... ... ... 2nd Ed. 1906

**Mummery, P. L.** ... *The Sigmoidoscope* ... ... ... ... ... ... ... ... ... 1906

**Oliver, G.** ... ... *Studies in Blood-Pressure* ... ... ... ... ... ... ... 1906

**Orton, H. Walsham and C. H.** *Röntgen Rays in Chest Diseases* ... ... ... 1906

**Phillips, J.** ... ... *Diseases of Women* ... ... ... ... ... ... ... 4th Ed. 1906

**Pilcher, R. B.** ... *A List of Official Chemical Appointments* ... ... ... 1906

**Ritchie, J. J. G. Brown and W. T.** *Medical Diagnosis* ... ... 5th Ed. 1906

**Robinson, M.** ... *A Guide to Urine Testing* ... ... ... ... 3rd Ed. 1906

**Rolleston, T. C. Allbutt and H. D.** [Eds.] *A System of Medicine.* Vol. II. Part I. ... ... ... ... [2nd Ed.] 1906

**Rose, F.** ... ... *The Use of Shower Baths in England* ... ... ... ... 1906

**Saundby, R.** ... *Treatment of Diseases of the Digestive System* ... ... 1906

**Savill, T. D.** ... *Neurasthenia* ... ... ... ... ... ... ... ... 3rd Ed. 1906

**Schlesinger, H.** ... *Indications for Operation in Disease of the Internal Organs.* (Tr. by K. W. Monsarrat) ... ... ... 1906

**Stacpoole, F.** ... *Women's Health* ... ... ... ... ... ... ... 2nd Ed. 1906

**Stewart, L.** ... ... *On Tendency to Disease of Body and Mind* ... ... (2) 1828

**Thomson and A. Miles, A.** *Manual of Surgery.* Vol. I. ... ... 2nd Ed. 1906

**Thomson, St. C.** ... *Submucous Excision of Deviations and Spurs of the Nasal Septum* ... ... ... ... ... ... ... ... ... 1906

**Védy, L.** ... ... *La Fièvre bilieuse hémoglobinurique dans le Bassin du Congo* ... ... ... ... ... ... ... ... ... 1907

**Vincent, R.** ... ... *Nutritional Disorders of Infancy* ... ... ... ... ... 1906

**Walsham and C. H. Orton, R.** *Röntgen Rays in Chest Diseases* ... ... ... 1906

**Westcott, W. H. Martindale and W. W.** *The Extra Pharmacopœia* 12th Ed. 1906

**Williams, L.** ... *Minor Maladies* ... ... ... ... ... ... ... ... 1906

*X-rays in Diagnosis* ... ... ... ... ... ... ... ... ... ... ... ... [1906]

**Yonge, E. S.** .. *Polypus of the Nose* ... ... ... ... ... ... ... 1906

### TRANSACTIONS, REPORTS, JOURNALS, &c.

American Journal of Obstetrics, The ... ... ... ... ... Vol. LIII. 1906

American Journal of Ophthalmology, The... ... ... ... Vol. XXII. 1905

American Journal of the Medical Sciences, The ... ... Vol. CXXXI. 1906

American Laryngological Association, Transactions of the ... ... ... 1906

| | | |
|---|---|---|
| American Medicine | Vol. XI. | 1906 |
| Archives of the Middlesex Hospital | (4) Vol. VIII. | 1906 |
| Association of American Physicians, Transactions of the | Vol. XX. | 1905 |
| Boston Medical and Surgical Journal, The | Vol. CLIV. | 1906 |
| Bristol Health Report, 1905 | | 1906 |
| British Gynæcological Journal, The | Vol. XXI. | 1905-06 |
| Buffalo Medical Journal | Vol. LXI. | 1906 |
| Bulletin de l'Institut international de Bibliographie | | 1904 |
| Canada Lancet, The | Vol. XXXIX. | 1905-06 |
| Cincinnati Lancet-Clinic, The | Vol. XCV. | 1906 |
| Clinical Journal, The | Vol. XXVII. | 1906 |
| Clinical Society's Transactions | Vol. XXXIX. | 1906 |
| Congrès de la Société internationale de Chirurgie, Procès-verbaux du 1er | | 1906 |
| Contributions from the Department of Neurology, Harvard Medical School | Vol. I. | 1906 |
| Deutsches Archiv für Geschichte der Medicin | (1) Bds. I.—IV. | 1878-81 |
| | VI. | 1883 |
| Giornale internazionale della Scienze mediche | | 1905 |
| Glasgow Medical Journal, The | Vol, LXV. | 1906 |
| Grant College Medical Society, Transactions of the | | 1906 |
| Grèce médicale, La | | 1905 |
| Henry Phipps Institute, 2nd Annual Report of the | | 1906 |
| Hospital, The | Vol. XXXIX. | 1906 |
| Hospitals—Philadelphia Hospital Reports | Vol. VI. | 1905 |
| Statistical Tables of St. Bartholomew's Hospital, 1905 | | 1906 |
| Journal of Medical Research, The | Vol. XIV. | 1905-06 |
| Journal of Obstetrics and Gynæcology, The | Vol. IX. ... | 1906 |
| Journal of the American Medical Association | Vol. XLVI. | 1906 |
| Library World, The | Vol. VIII. | 1906 |
| Louisville Monthly Journal, The | Vol. XII. | 1905-06 |
| Medical Library and Historical Journal | Vol III. | 1905 |
| Medical Press and Circular, The | [Vol. CXXXII.] | 1906 |
| Medical Record | Vol. LXIX. | 1906 |
| Medico-Chirurgical Transactions | Vol. LXXXIX. | 1906 |
| Münchener medizinische Wochenschrift | Bd. I. für | 1906 |
| Obstetrical Transactions | Vol. XLVII. | 1906 |
| Philosophical Transactions, Volume of Reprints from | (3) | [c. 1800-44] |
| Practitioner, The | [Vol. LXXVI.] | 1906 |
| Progressive Medicine | Vol. III. | 1906 |
| Public Health | Vol. XVIII. | 1905–06 |
| Publishers' Circular, The | (5) Vols. LXXXI., 1904, LXXXIV. | 1906 |
| St. Louis Medical Review | Vols. LI., 1905, LIII. | 1906 |
| Sanitary Record, The | Vol. XXXVII. | 1906 |
| Sei-I-Kwai Medical Journal | Vols. VI., 1887, XXIV. | 1905 |
| Society of Anæsthetists, Transactions of the | Vol. VII. | 1905 |

### Local Medical Notes.

**University College, Bristol.**—The annual prize distribution of the Faculty of Medicine took place on October 2nd, when Professor Alexander MacAlister, of Cambridge, distributed the prizes and delivered an address. The report of the Faculty showed how successful the students of the Medical Faculty have been in the various examinations, and they also recorded their regret at losing the services of Professor Travers.

The dinner of the past and present students of the College was held in the evening, under the presidency of Dr. F. J. Wethered; the guest of the evening was Professor MacAlister.

The Medical Entrance Scholarship (of the value of £75) has been awarded to Mr. R. F. Bolt.

Students of the College have recently passed the following examinations:—

CONJOINT BOARD OF THE ROYAL COLLEGES OF PHYSICIANS AND SURGEONS.—*Chemistry and Physics:* L. C. Watkins-Baker. *Biology:* I. R. Hudleston. *Practical Pharmacy:* E. R. Sircom. *Medicine, Surgery:* G. S. Parkinson. *Midwifery:* J. W. J. Willcox, A. E. Iles, E. V. Connellan, P. S. Connellan.

**Bristol Royal Infirmary.**—The following appointment has been made:—*Casualty Officer:* V. B. Green-Armytage, M.R.C.S., L.R.C.P.

**Bristol General Hospital.**—The following appointments have been made:—*House Surgeon:* W. J. H. Pinniger, M.B., B.S. Lond. *House Physician:* F. G. Bergin, M.R.C.S., L.R.C.P. *Casualty Officer:* M. R. O. Wilson, M.R.C.S., L.R.C.P. *Assistant House Physician:* J. F. Blackett, M.B., B.S. Lond.

**Stanley Hospital, Liverpool.**—C. A. Moore, M.B., B.S. Lond., has been appointed House Surgeon.

**Southern Hospital, Liverpool.**—F. F. Leighton, M.B., B.C. Cantab., has been appointed Resident Pathologist and Registrar.

**General Hospital, Birmingham.**—J. H. Board, M.R.C.S., L.R.C.P., has been appointed House Surgeon.

**Leicester Infirmary.**—G. S. Parkinson, M.R.C.S., L.R.C.P., has been appointed Junior House Surgeon.

**Newport Hospital.**—R. E. Thomas, M.R.C.S., L.R.C.P., has been appointed House Surgeon.

**Royal Hospital, Sheffield.**—W. W. King, M.R.C.S., L.R.C.P., has been appointed Junior House Surgeon.

**Shepton Mallet.**—D. T. Price, M.B. Lond., has been appointed Medical Officer to the Board of Guardians.

**Visits of Professor Leishman and Dr. Bolton.**—On the invitation of Prof. Walker Hall, a number of those interested in research work gathered together at the Bristol Eye Hospital to meet Professor Leishman, on Saturday, October 20th. After an informal reception, some fine specimens illustrating benign and malignant malarial fevers and relapsing fevers were demonstrated and inspected. Among these were some beautifully-stained rosette forms, young rings, and Schüffner's clots. A smear from brain tissue showing a capillary crowded with deeply-staining rosettes called forth universal admiration, as did also a brilliant example of Levaditi's method for staining spirochætes in tissues. Professor Leishman then answered innumerable questions concerning his well-known staining method, and the theories and bypaths of protozoal infections. Later the company, tired of standing, sat in a ring around the Professor, and listened to a ten minutes' talk upon the method and value of inoculation for typhoid fever. Following this more queries were dealt with, and the meeting terminated with a description of the Leishman method for counting white blood corpuscles in stained films.

On Thursday, November 29th, about forty medicals accepted the invitation to meet Dr. Charles Bolton, of University College, London. As in the case of the previous meeting, the Committee of the Bristol Eye Hospital kindly lent their rooms for the purpose. After the introductions, Dr. Bolton gave a most interesting account—illustrated by micro- and lantern slides—of his researches upon "gastro-toxins." Gastro-toxins are cytolytic in nature, and are formed in response to the injection of gastric cells into animals. After the injections, patches of necrosis, hemorrhage and ulceration appear in the mucous membrane of the stomach. The gastro-toxins are hæmolytic, are able to agglutinate cell granules, and produce hyaline changes in gastric cells. They appear to be specific in action.

### INDEX.

(R.) = Review.

Abdominal wall, Fibroma of the—J. L. Firth, 304.
Aberdeen university quater-centenary, 374.
Acland, Dr. T. D.—On the hours of sleep at public schools (R.), 164.
Actinomycosis, The biology of the micro-organism of—Dr. J. H. Wright (R.), 67.
Adams-Stokes affection of the heart, 143.
Allbutt, Dr. T. C.—On professional education, with special reference to medicine (R.), 368.
Allbutt and H. D. Rolleston, Drs. T. C.—System of medicine (R.), 363.
Alveolar cleft palate—Dr. E. Fawcett, 237.
American Dermatological Association, Transactions of the (R.), 64.
American Surgical Association, Transactions of the (R.), 64.
Aortic insufficiency, 254.
Appendicitis, 145.
Appendicitis—C. B. Lockwood (R.), 367.
Arrhythmia, 251, 252.

Bacteriology and hæmatology, Clinical—Dr. W. D'E. Emery (R.), 266.
Bain and W. Edgecombe, Drs. W.—The physiology and therapeutics of the Harrogate waters, baths and climate (R.), 166.
Bardswell, Dr. N. D.—The consumptive working-man (R.), 365.
Barrett, Dr. A. W.—Dental surgery (R.), 166.
Barton, Dr. G. A. H.—A guide to the administration of ethyl chloride (R.), 267.
Biliary cirrhosis, Case of splenomegalic—Dr. E. C. Williams, 42.
Blood immunity and blood relationship—Dr. G. H. F. Nuttall, (R.), 157.
Bristol, Health notes, 1905—Dr. D. S. Davies, 137.
Bristol Medico-Chirurgical Society, 88, 188, 382.
Bristol Royal Infirmary, 70.
British Electro-Therapeutic Society, 177, 276.
British Medical Association, Toronto meeting, 268.
Broadbent, Sir W. H. and Dr. J. F. H.—Heart disease (R.), 364.
Bruce, Dr. J. M.—Materia medica and therapeutics (R.), 165.
Bulkley, Dr. L. D.—On the relations of diseases of the skin to internal disorders (R.), 157.

Cancer, The nature and treatment of—Dr. J. A. Shaw-Mackenzie (R.), 165.
Carbohydrate metabolism and diabetes—Dr. F. W. Pavy (R.), 359.
Cardiac rhythm, 251.
Carless, Dr. W. Rose and A.—Manual of surgery (R.), 68.
Carson, H. W.—Aids to surgical diagnosis (R.), 368.
Carwardine, T.—Report on surgery, 254.
Catalogue (Index-) of the library of the Surgeon-General's office, United States Army (R.), 166.
Cataract in the capsule, Extraction of, 258.
Cerebro-spinal fever, 176.
Chance, Dr. E. J.—Bodily deformities (R.), 265.
Chaplin, Drs. E. H. Colbeck and A.—The science and art of prescribing (R.), 169.
Charles, Dr. J. R—On the treatment of diabetes with secretin, 218; Some effects of fright in medicine, 23.
Chemistry, A manual of—Dr. A. P. Luff and F. J. M. Page (R.), 61.

Children, Surgical diagnosis and treatment of cases associated with vomiting in—Dr. J. Swain, 116.
Clarke, Dr. J. M.—Report on medicine, 249.
Cleft palate, Alveolar—Dr. E. Fawcett, 237.
Climatic treatment, Handbook of—Dr. W. R. Huggard (R.), 160.
Clinical methods—Dr. R. Hutchison and H. Rainy (R.), 163.
Clinical pathology, New methods of —Dr. I. W. Hall, 121.
Cohn, Dr. T. — Die palpablen gebilde des normalen menschlichen körpers und deren methodische palpation (R.), 265.
Colbeck and A. Chaplin, Drs. E. H.—The science and art of prescribing (R.), 169.
Colitis polyposa—Dr. C. Coombs, 31.
College of Physicians and Surgeons of Columbia University, Studies from the department of pathology of the (R.), 368.
Collins, Obituary notices of Henry William, 94.
Consumption: treatment at home and rules for living—Dr. H. W. Crowe (R.), 364.
Consumptive working-man, The — Dr. N. D. Bardswell (R.), 365.
Coombs, Dr. C.—Colitis polyposa, 31; Report on medicine, 344.
Cornea, Pneumococci in diseases of the, 261.
Coroner, The King's—R. H. Wellington (R.), 266.
Crowe, Dr. H. W.—Consumption: treatment at home and rules for living (R.), 364.

Davies, Dr. D. S.—Health notes in Bristol, 1905, 137; The adaptation of means in the prevention of disease, 193.
Deformities, Bodily—E. J. Chance (R.), 265.
Dental surgery—Dr. A. W. Barrett (R.), 166.
Dental surgery, Golden rules of—C. W. Glassington (R.), 63.
Dermatitis from without and dermatitis from within—Dr. A. J. Harrison, 325.
Dermatology, Reports on—Dr. J. A. Nixon, 353; Dr. W. K. Wills, 57.
Development impeded by defective nasal respiration—Dr. P. W. Williams, 17.
Diabetes, Carbohydrate metabolism and—Dr. F. W. Pavy (R.), 359.
Diabetes mellitus—Dr. C. v. Noorden (R.), 261.
Diabetes treated with secretin—Dr. J. R. Charles, 218.
Diagnosis, Gynæcological—Dr. A. E. Giles (R.), 170.
Diagnosis, Surgical—H. W. Carson (R.), 368.
Diet, Effect of, on the fat-secretion of the skin, 353.
Dietetics and disease—Dr. J. S. Wallace (R.), 159.
Digalen, 250.
Digitalis, Cumulative action of, 249.
Disease, The adaptation of means in the prevention of—Dr. D. S. Davies, 193.
Dispensing made easy—Dr. W. G. Sutherland (R.), 162.
Diver's paralysis, 144.
Doctor as expert and pioneer, The —Dr. C. J. Whitby, 222.
Drink restriction, particularly in obesity—Drs. C. v. Noorden and H. Salomon (R.), 163.
Dunbar, Dr. Eliza L. W.—The new theory and prophylaxis of puerperal eclampsia, 132.

Eclampsia, Puerperal—Dr. E. L. W. Dunbar, 132.
Edgecombe, Drs. W. Bain and W. —The physiology and therapeutics of Harrogate waters, baths and climate (R.), 166.
Edgeworth, Dr. F. H.—Notes of a case of acute yellow atrophy of the liver, 37.
Edinburgh medical journal, The (R.), 65.
Editorial notes, 70, 171, 268, 370.
Education, Professional—Dr. T. C. Allbutt (R.), 368.
Egypt, The medical diseases of—Dr. F. M. Sandwith (R.), 162.
Electrical methods in affections of the stomach and intestines—Dr. G. Herschell (R.), 69.
Electricity, The uses of static—Dr. P. King, 233.
Emery, Dr. W. D'E. — Clinical bacteriology and hæmatology (R.), 266.
Encyclopedia and dictionary of medicine and surgery, Green's (R.), 363.

Epithelioma of tongue, Case of—C. H. Whiteford, 45.
Ethyl chloride, A guide to the administration of—Dr. G. A. H. Barton (R.), 267.
Exercises in the treatment of tabetic ataxia—Dr. M. Faure, 210.

Fat-secretion of the skin, Effect of diet on the, 353.
Faure, Dr. M.—The treatment of tabetic ataxia by methodical exercises, 210.
Fawcett, Dr. E.—The explanation of alveolar cleft palate, 237.
Fibroma of the abdominal wall—J. L. Firth, 304.
First aid to the injured—Drs. F. J. Warwick and A. C. Tunstall (R.), 267.
Firth, Dr. J. L.—Fibroma of the abdominal wall, 304; Report on surgery, 52.
Fitzpatrick, Obituary notice of William, 287.
Flemming, C. E. S.—Lumbago, 110.
Food factor in disease—Dr. F. Hare (R.), 160.
Fortescue-Brickdale, Dr. J. M.—Anti-tuberculous vaccines and sera, 1; Proteins and protein metabolism, 318.
Fowler, Obituary notice of Richard Sumner, 390.
Fox lecture, The Long—Dr. A. J. Harrison, 325.
Fraser, Obituary notice of G. B., 286.
French, Dr. H.—Medical laboratory methods and tests (R.), 167.
Fright in medicine, Some effects of—Dr. J. R. Charles, 23.

Gastro-enterostomy, 254.
Gibson, Dr. G. A.—The nervous affections of the heart (R.), 163.
Giles, Dr. A. E. — Gynæcological diagnosis (R.), 170.
Glasgow, Public health administration in—Dr. J. B. Russell (R.), 170.
Glassington, C. W.—Golden rules of dental surgery (R.), 63.
Graves's disease, 47.
Green's encyclopedia and dictionary of medicine and surgery (R.), 363.
Groves, Dr. E. W. H.—The relation of the pelvic floor to pelvic displacements and pain in the female, 97.
Guy's hospital reports (R.), 63.
Gynæcological diagnosis—Dr. A. E. Giles (R.), 170.

Haegler, Dr. C. S.—The cleansing, disinfection, and protection of the hands (R.), 158.
Hall, Dr. I. W.—New methods of clinical pathology, 121.
Hands, The cleansing, disinfection, and protection of the—Dr. C. S. Haegler (R.), 158.
Hare, Dr. F.—The food factor in disease (R.), 160.
Harrison, Dr. A. J.—Dermatitis from without and dermatitis from within, 325.
Harrison, R.—The urethrotomies and kidney capsulotomy (R.), 367.
Harrogate waters, baths, and climate, The physiology and therapeutics of the—Drs. W. Bain and W. Edgecombe (R.), 166.
Heart affections of scarlet fever, 253.
Heart disease—Sir W. H. and Dr. J. F. H. Broadbent (R.), 364.
Heart, "Nauheim" treatment of diseases of—Dr. L. Thorne-Thorne (R.), 365.
Heart, Nervous affections of the——Dr. G. A. Gibson (R.), 163.
Heat-regulation in universal skin diseases, 355.
Hemorrhage, Treatment of internal, 140.
Herschell, Dr. G. — Electrical methods in the treatment of affections of the stomach and intestines (R.), 69; Indigestion (R.), 67.
Huggard, Dr. W. R—A handbook of climatic treatment, including balneology (R.), 160.
Hurst, Dr. A. M. Marshall and C. H.—A junior course of practical zoology (R.), 66.
Hutchison and H. Rainey, Dr. R.—Clinical methods (R.), 163.
Hygiene and public health—Drs. B. A. Whitelegge and G. Newman (R.), 167.

Indigestion—Dr. G. Herschell (R), 67.
Infant feeding, Sodium citrate in, 344.
Infections, Hints on the management of the commoner—Dr. R.W. Marsden, (R.), 366.

Insane, Plea for the more energetic treatment of the—C. Williams (R.), 68.
International medical congress at Lisbon, 173.

Jellett, Dr. H.—A manual of midwifery (R.), 263.

Kaminer, Drs. H. Senator and S.—Health and disease in relation to marriage and the married state (R.), 262.
Kerr, Obituary notice of J. G. Douglas, 388.
Kidney capsulotomy, The urethrotomies and—R. Harrison (R.), 367.
Kidney, Tubercular disease of the, 52.
King, Dr. P.—The uses of static electricity as an aid to recovery, 233.

Laboratory reports of the Royal College of Physicians, Edinburgh (R.), 169.
Library of the Bristol Medico-Chirurgical Society, 85, 185, 284, 379.
Life, Means for the prolongation of —Sir H. Weber (R.), 168.
Lister Institute of preventive medicine. Collected papers (R.), 63.
Liver, Acute yellow atrophy of—Dr. B. M. H. Rogers, 40; Dr. F. H. Edgeworth, 37.
Loane, M.—Simple introductory lessons in midwifery (R.), 369.
Local medical notes, 95, 192, 287, 391.
Lockwood, C. B.—Appendicitis (R.), 367.
Lucas, Dr. J. J. S.—The prognosis of pulmonary tuberculosis, 12.
Luff and J. F. M. Page, Dr. A. P.—A manual of chemistry (R.), 61.
Lumbago—C. E. S. Flemming, 110.
Lupus erythematosus, 57.

Magnet in ophthalmology, Use of giant, 258.
Malignant disease of the vermiform appendix, 256.
Marriage, Health and disease in relation to—Drs. H. Senator and S. Kaminer (R.), 262.
Marsden, Dr. R. W.—Hints on the management of the commoner infections (R.), 366.
Marshall and C. H. Hurst, Dr. A. M.—A junior course of practical zoology (R.), 66.
Materia medica and therapeutics—Dr. J. M. Bruce (R.), 165.
Medical annual (R.), 365.
Medical practice, Golden rules of—Dr. L. Smith (R.), 66.
Medical Society's transactions (R.), 165.
Medicine, Reports on—Dr. J. M. Clarke, 249; Dr. C. Coombs, 344; Dr. G. Parker, 140; Dr. R. S. Smith, 47.
Medicine, System of—Drs. T C. Allbutt and H. D. Rolleston (R.), 363.
Medicine, The theory and practice of—Dr. F. J. Roberts (R.), 163.
Merck's annual report (R.), 66.
Mesenteric thrombosis, A case of—G. M. Smith, 216.
Metabolism, Proteins and protein—Dr. J. M. Fortescue-Brickdale, 318.
Midwifery, A manual of—Dr. H. Jellett (R.), 263.
Midwifery, Simple lessons in—M. Loane (R.), 369.
Mole, H. F.—Report on surgery, 348.
Morton, C. A.—Report of surgery, 145; The preservation of the limb in the treatment of sarcoma of bone, 308.
Museums Association, 277.
Mycosis fungoides, Pathology of, 356.

Nasal respiration, Development impeded by defective—Dr. P. W. Williams, 17.
National League for Physical Education and Improvement, 79.
"Nauheim" treatment of chronic diseases of the heart—Dr. L. Thorne-Thorne (R.), 365.
Nephrectomy in tubercular disease of the kidney, 52.
Nervous affections of the heart—Dr. G. A. Gibson, (R.), 163.
Newman and B. A. Whitelegge, Drs. G.—Hygiene and public health (R.), 167.
Newport Medical Society, 190.
Nixon, Dr. J. A.—Report on dermatology, 353.
Noorden and H. Salomon, Drs. C. v.—Drink restriction, particularly in obesity (R.), 163.

Noorden, Dr. C. v. — Diabetes mellitus (R.), 261.
Nursing of consumption, The modern—Dr. Jane Walker (R.), 66.
Nuttall, Dr. G. H. F. — Blood immunity and blood relationship (R.), 157.

Obesity, Drink restriction in—Drs. C. v. Noorden and H. Salomon (R.), 163.
Obituary, 93, 191, 286, 287, 386.
Obstetrics, Report on—Dr. W. C. Swayne, 151.
Ophthalmology, Report on—Dr. C. H. Walker, 258.

Page, Dr. A. P. Luff and J. F. M. —A manual of chemistry (R.), 61.
Palpation, Die palpablen gebilde des normalen menschlichen körpers und deren methodische—Dr. T. Cohn (R.), 265.
Parker, Dr. G.—Report on medicine, 140.
Parry, Obituary notice of J. H., 191.
Parsons, Obituary notice of F. J. C., 93.
Pathology, New methods of clinical —Dr. I. W. Hall, 121.
Pavy, Dr. F. W.—Carbohydrate metabolism and diabetes (R.), 359.
Pelvic displacements and pain, Relation of the pelvic floor to— Dr. E. W. H. Groves, 97.
Pericardium, Adherent, 250.
Peritonitis, Transfusion of saline solution in, 147.
Philadelphia, Transactions of the College of Physicians of (R.), 65, 366.
Physiology, Manual of—Dr. G. N. Stewart (R.), 168.
Pneumococci in diseases of the cornea, 261.
Prescribing, The science and art of —Drs. E. H. Colbeck and A. Chaplin (R.), 169.
Preservation of the limb in the treatment of sarcoma of bone— C. A. Morton, 308.
Prolongation of life, On means for the—Sir H. Weber (R.), 168.
Proteins and protein metabolism— Dr. J. M. Fortescue-Brickdale, 318.
Psoriasis, Its connection with seborrhœa, 57.
Public health administration in Glasgow—Dr. J. B. Russell (R.), 170.
Puerperal eclampsia—Dr. Eliza L. W. Dunbar, 132.
Pulmonary tuberculosis, Prognosis of—Dr. J. J. S. Lucas, 12.

Rainey, Dr. R. Hutchinson and H. —Clinical methods (R.), 163.
Ringworm treated by X-rays, 58.
Roberts, Dr. F. J.—The theory and practice of medicine (R.), 163.
Rogers, Dr. B. M. H.—A case of acute yellow atrophy of the liver in a child, 40.
Rolleston, Drs. T. C. Allbutt and H. D.—System of medicine (R.), 363.
Rose and A. Carless, Dr. W.— Manual of surgery (R.), 68.
Royal Sanitary Institute, The, 78, 178, 270.
Russell, Dr. J. B.—Public health, administration in Glasgow (R.), 170.

Saline solution in peritonitis, Transfusion of, 147.
Salomon, Drs. C. v. Noorden and H.—Drink restriction (R.), 163.
Sanatoria for consumptives, 70.
Sanatorium, Life in a, 71.
Sandwith, Dr. F. M.—The medical diseases of Egypt (R.), 162.
Sanitary inspector's handbook— A. Taylor (R.), 63.
Sarcoma, inoperable, treated by injections of erysipelas streptococcus toxines, 148.
Sarcoma of bone, Preservation of the limb in the treatment of—C. A. Morton, 308.
Scarlet fever, Heart affections of, 253.
Seborrhœa, Its connection with psoriasis, 57.
Secretin in the treatment of diabetes—Dr. J. R. Charles, 218.
Senator and S. Kaminer, Drs. H.— Health and disease in relation to marriage and the marriage state (R.), 262.
Sera, Anti-tuberculous vaccines and —Dr. J. M. Fortescue-Brickdale, 1.
Sex and character—O. Weininger (R.), 360.
Shaw-Mackenzie, Dr. J. A.—The nature and treatment of cancer (R.), 165.

Sick, Preparations for the, 81, 179, 281, 376.
Skin diseases, Heat-regulation in universal, 355.
Skin, Effect of diet on the fat-secretion of the, 353.
Skin, Relation of diseases of, to internal disorders—Dr. L. D. Bulkley (R.), 157.
Sleep at public schools—Dr. T. D. Acland (R.), 164.
Smith, Dr. L.—Golden rules of medical practice (R.), 66.
Smith, Dr. R. S.—Report on medicine, 47.
Smith, G. M.—A case of mesenteric thrombosis, 216.
Smithsonian report (R.), 369.
Societies, Bristol Medico-Chirurgical, 88, 188, 382; British Electro-Therapeutic, 177, 276; Newport Medical, 190.
Sodium citrate in infant feeding, 344.
Splenomegalic biliary cirrhosis, Case of—Dr. E. C. Williams, 42.
Starch and infant feeding, 346.
Static electricity, Uses of—Dr. P. King, 233.
Stewart, Dr. G. N.—A manual of physiology (R.), 168.
Stomach, Electrical methods in affections of the—Dr. G. Herschell (R.), 69.
Stone in the ureter, 348.
Suppuration in female pelvis, 151.
Surgery, Manual of—Dr. W. Rose and A. Carless (R.), 68.
Surgery, Reports on—T. Carwardine, 254; J. L. Firth, 52; H. F. Mole, 348; C. A. Morton, 145.
Surgical diagnosis—H. W. Carson (R.), 368.
Sutherland, Dr. W. G.—Dispensing made easy (R.), 162.
Swain, Dr. J.—Difficulties in the surgical diagnosis and treatment of cases associated with vomiting in children, 116.
Swayne, Dr. W. C.—Report on obstetrics, 151.
Syphilis and yaws, Similarity between, 60, 61.
Syphilis, Inherited, 347.
Syphilis, Spirochæta of, 58.

Tabetic ataxia, Treatment by methodical exercises—Dr. M. Faure, 210.
Taylor, A. — Sanitary inspector's handbook (R.), 63.
Taylor, J.—X-ray therapeutics, 289.
Tests, Medical laboratory methods and—Dr. H. French (R.), 167.
Therapeutics, X-ray—J. Taylor, 289.
Thorne-Thorne, Dr. L. — The "Nauheim" treatment of chronic diseases of the heart (R.), 365.
Tongue, Epithelioma of the—C. H. Whiteford, 45.
Toxic amblyopia, 260.
Toxines, Treatment of inoperable sarcoma by injections of, 148.
Tubal pregnancy—C. H. Whiteford, 126.
Tuberculous vaccines and sera—Dr. J. M. Fortescue-Brickdale, 1.
Tunstall, Drs. F. J. Warwick and A. C.—"First Aid" (R.), 277.

University College, Bristol, Faculty of Medicine, Prize distribution, 370, 391.
University College Colston Society, 171.
University for Bristol, The proposed, 76, 171, 372.
Ureter, Stone in the, 348.
Urethrotomies and kidney capsulotomy, The—R. Harrison (R.), 367.

Vermiform appendix, Malignant disease of the, 256.
Vomiting in children, Surgical diagnosis and treatment of cases associated with—Dr. J. Swain, 116.

Walker, Dr. C. H.—Report on ophthalmology, 258.
Walker, Dr. Jane—The modern nursing of consumption (R.), 66.
Wallace, Dr. J. S.—The rôle of modern dietetics in the causation of disease (R.), 159.
Warwick and A. C. Tunstall, Drs. F. J.—"First aid" (R.), 267.
Wathen, Obituary notice of John Hancocke, 386.
Waugh, Obituary notice of Alexander, 389.
Weber, Sir H.—On means for the prolongation of life (R.), 168.
Weininger, O.—Sex and character (R.), 360.
Wellington, R. H.—The King's coroner (R.), 266.
Whitby, Dr. C. J.—The doctor as expert and pioneer, 222.

Whiteford, C. H.—Clinical note on a case of epithelioma of tongue in a young woman, 45; Tubal pregnancy—its diagnosis and treatment, 126.
Whitelegge and G. Newman, Drs. B. A.—Hygiene and public health (R.), 167.
Williams, C.—A plea for the more energetic treatment of the insane (R.), 68.
Williams, Dr. E. C.—Notes on a case of splenomegalic biliary cirrhosis in a boy aged six years, 42.
Williams, Dr. P. W.—Why defective nasal respiration impedes growth and development, 17.
Wills, Dr. W. K.—Report on dermatology, 57.
Winsley sanatorium, 278.
Working-man, The consumptive—Dr. N. D. Bardswell (R.), 365.
Wright, Dr. J. H.—The biology of the micro-organism of actinomycosis (R.), 67.

X-rays and the thyroid gland, 51.
X-rays, Ringworm treated by, 58.
X-ray therapeutics—J. Taylor, 289.

Yaws, Similarity between syphilis and, 60, 61.
Yellow atrophy of the liver, Cases of acute—Dr. F. H. Edgeworth, 37; in a child—Dr. B. M. H. Rogers, 40.

Zoology, Practical—Dr. A. M. Marshall and C. H. Hurst (R.), 66.

J. W. Arrowsmith, Printer, Quay Street, Bristol.

